# Navigating weight, risk and lifestyle conversations in maternity care: a qualitative study among pregnant women with obesity

**DOI:** 10.1186/s12884-024-06751-1

**Published:** 2024-08-23

**Authors:** Heidi L. Sandsaeter, Trine Tetlie Eik-Nes, Linn Okkenhaug Getz, Elisabeth Balstad Magnussen, Janet W. Rich-Edwards, Julie Horn

**Affiliations:** 1 Department of Public Health and Nursing, Norwegian University of Science and Technology, Trondheim, Norway; 2 Department of Obstetrics and Gynecology, Levanger Hospital, Nord-Trøndelag Hospital Trust, Levanger, Norway; 3 Department of Neuromedicine and Movement Science, Norwegian University of Science and Technology, Trondheim, Norway; 4 Stjørdal Community Mental Health Centre, Levanger Hospital, Levanger, Norway; 5 Research Unit for General Practice, Department of Public Health and Nursing, Norwegian University of Science and Technology, Trondheim, Norway; 6 Department of Obstetrics and Gynecology, St. Olav’s University Hospital, Trondheim, Norway; 7 Department of Clinical and Molecular Medicine, Norwegian University of Science and Technology, Trondheim, Norway; 8 Division of Women’s Health and Connors Center for Women’s Health and Gender Biology, Department of Medicine, Brigham and Women’s Hospital, Boston, MA USA; 9 Department of Epidemiology, Harvard T.H. Chan School of Public Health, Boston, MA USA

**Keywords:** Pre-pregnancy obesity, Insider perspective, Weight stigma, Shame avoidance strategy, Maternity care

## Abstract

**Background:**

Pregnant women with obesity face heightened focus on weight during pregnancy due to greater risk of medical complications. Closer follow-up in maternety care may contribute to reduce risk and promote health in these women. The aim of this study was to gain a deeper insight in how pregnant women with obesity experience encounters with healthcare providers in maternity care. How is the received maternity care affected by their weight, and how do they describe the way healthcare providers express attitudes towards obesity in pregnancy?

**Methods:**

We conducted in-depth interviews with 14 women in Trøndelag county in Norway with pre-pregnancy BMI of ≥ 30 kg/m^2^, between 3 and 12 months postpartum. The study sample was strategic regarding age, relationship status, education level, obesity class, and parity. Themes were developed using reflexive thematic analysis. The analysis was informed by contextual information from a prior study, describing the same participants’ weight history from childhood to motherhood along with their perceptions of childhood quality.

**Results:**

This study comprised of an overarching theme supported by three main themes. The overarching theme, Being pregnant with a high BMI: a vulnerable condition, reflected the challenge of entering maternity care with obesity, especially for women unprepared to be seen as “outside the norm”. Women who had grown up with body criticism and childhood bullying were more prepared to have their weight addressed in maternity care. The first theme, Loaded conversations: a balancing act, emphasizes how pregnant women with a history of body criticism or obesity-related otherness proactively protect their integrity against weight bias, stigma and shame. The women also described how some healthcare providers balance or avoid weight and risk conversations for the same reasons. Dehumanization: an unintended drawback of standardized care makes apparent the pitfalls of prioritizing standardization over person-centered care. Finally, the third theme, The ambivalence of discussing weight and lifestyle, represent women’s underlying ambivalence towards current weight practices in maternity care.

**Conclusions:**

Our findings indicate that standardized weight and risk monitoring, along with lifestyle guidance in maternity care, can place the pregnant women with obesity in a vulnerable position, contrasting with the emotionally supportive care that women with obesity report needing. Learning from these women’s experiences and their urge for an unloaded communication to protect their integrity highlights the importance of focusing on patient-centered practices instead of standardized care to create a safe space for health promotion.

**Supplementary Information:**

The online version contains supplementary material available at 10.1186/s12884-024-06751-1.

## Background

Obesity has for several decades been increasing in all age groups in much of the world [1, 2]. This condition not only increases the risk of developing complications during pregnancy and childbirth, but also negatively affects the future health of both the mother and child [3–5]. A recent meta-analysis of nearly 200 000 participants reported that the absolute risk for any adverse pregnancy outcome (preeclampsia, gestational hypertension, gestational diabetes, cesarean delivery, preterm birth, and small or large size for gestational age) was 61% among women with obesity class 3 (BMI ≥ 40 kg/m^2^), compared to 34% among women categorized as normal weight. Pre-pregnancy obesity thus represents a major challenge in maternity care [6].

Intensified care and monitoring of this group of pregnant women in order to prevent, detect and treat pregnancy complications at an early stage and assess the need for lifestyle advice has therefore been proposed in several international guidelines for pregnancy care [7, 8]. Knowledge of associated risks have been assumed to encourage more health-promoting behavior [9–11], and enhanced care therefore centers on the woman’s weight and lifestyle advice during pregnancy. However, pregnant women’s ability to translate increased knowledge about risks of complications and diseases into behavioral change depends on many factors, including how risk is communicated to the woman [12, 13]. Healthcare providers face challenges in communication related to obesity due to fear of inducing shame, limited time, limited expertise on how to address weight during pregnancy and low trust in the effectiveness of lifestyle counseling [14–18]. Providers may experience a dilemma when following guidelines focusing on weight as they seek to avoid weight stigmatization and inducing shame. Consequently, strict adherence to standard care procedures based on body mass index is avoided by some providers to prevent weight stigma and shame in the patient relationship [16, 19].

Women, especially those with BMI levels above 35 kg/m^2^, are the most affected by weight stigma [20, 21]. Weight stigma and its consequences have been described as “a social devaluation and denigration of individuals because of their excess body weight, and can lead to negative attitudes, stereotypes, prejudice, and discrimination” [22]. Perceived weight stigma has been associated with increased negative emotions, poorer quality of life, worse health and increased risk of premature death [22, 23]. Unfortunately, weight stigma is found to be common among healthcare providers [21, 24, 25], and discrimination against patients with obesity in maternity care has been described in several studies [26–29]. Experiences with weight stigma in healthcare encounters increase the likelihood of unhealthy eating habits and reduced physical activity with subsequent weight gain and obesity [9, 30].

Obesity is a multifactorial condition with strong evidence suggesting associations with adverse childhood experiences (ACEs), life trauma and unbuffered stress [31–34]. The positive association between ACEs and pre-pregnancy obesity has been described in several studies [35–38], with the strongest associations for women with class 3 obesity [39]. Prior experiences with ACEs can render individuals more vulnerable when faced with a weight focused and standardized pregnancy follow-up. Knowledge of the woman’s childhood background can increase healthcare providers’ understanding of relationships between higher weight, ACEs, and stress, and thus reduce weight stigma. It can therefore be considered a key element of care for women with pre-pregnancy obesity. However, the role of childhood quality has been rarely considered in previous studies of women with obesity receiving maternity care.

In a previous qualitative study, we explored the weight history from childhood to motherhood of 14 women with pre-pregnancy obesity in the context of their childhood quality [40]. In the same population, we now aim to use this knowledge of the subjective experiences of these women’s weight history to enhance our understanding of how childhood quality affects the experiences of women with pre-pregnancy obesity in maternity care. To our knowledge, these experiences have not been explored in previous studies. We also seek to understand in what ways women with pre-pregnancy obesity in Norway feel that their care is affected by weight stigma, and how they describe attitudes towards obesity among clinicians. This knowledge can reveal enabling and constraining factors for monitoring and supportive care of weight-related risks in pregnancy and postpartum from the women’s perspective.

## Method

We employed a qualitative approach to gather the perceptions and experiences of women with pre-pregnancy obesity in standard maternity care in Norway as this is a suitable method to describe and interpret subjective experiences [41]. All research team members have prior experience in the field and varied professional backgrounds: a midwife (HLS), two obstetrician-gynecologists (JH and EBM), a researcher with a mental healthcare background specializing in obesity and eating disorders (TTEN), a physician and professor of behavioral sciences in medicine (LOG) and an epidemiologist (JWRE). This collaborative effort is believed to have enriched the research by broadening perspectives, mitigating blind spots, and enhancing awareness of our preconceptions.

Stakeholders from the Trondheim branches of the user organizations National Association for People with Overweight, the Centre against Incest and Sexual Abuse Nord-Trøndelag, and the Norwegian Women’s Public Health Association (Levanger youth branch) provided valuable input in the creation of the interview guide, the information sheet for this study and specific recommendations for conducting the in-depth interviews in a considerate and supportive manner.

The study was approved by the Central Norway Regional Committee for Medical and Health Research Ethics (reference number 13.04.21/222481). All participants provided written informed consent.

### Sampling and recruitment

In Norway, all ante- and perinatal care is provided free of charge. Pregnant women may choose between care provided by a family physician, a midwife, or a combination. Additionally, pregnant women with obesity (BMI *≥* 30 kg/m^2^) and comorbidities or a BMI ≥ 35 kg/m^2^ are offered extra monitoring and follow up at the nearest hospital maternity ward. Moreover, they are advised to limit gestational weight gain to 5–9 kg through enhanced lifestyle follow-up based on national guidelines for the general population [7].

Potential study participants were women with pre-pregnancy obesity receiving obstetric care at one of the three hospitals offering this in Trøndelag County. Women were identified through the databases of St. Olavs Hospital (an urban university hospital), Levanger Hospital (a large local hospital) or Namsos Hospital (a small local hospital) using the International Classification of Diseases (ICD-10) codes indicating maternity care for pre-pregnancy obesity.

After identification of women in the hospital databases, invitation letters containing study information were sent to 137 potential participants 3–12 months postpartum. Those interested in participating (15 women) sent an SMS or email to the first author (HLS), and were then contacted by telephone to arrange a suitable place and time for an interview. A brief SMS was sent to all participants the day before the interview to confirm the appointment. 14 women (10% of those invited) were included in the study. The invitation to participate in the study was sent out in waves. Between each wave, new interviews were conducted. The inclusion of new participants was guided by an ongoing assessment of the data’s information power to address the research questions of the study [42].

### Interviews

Before the interviews, women’s sociodemographic and maternity/pregnancy characteristics were gathered using a questionnaire. Participants gave birth between December 2020 and April 2022, and were 18 years or older and proficient in either Norwegian or English. The final study sample was strategic in relation to key factors that could be expected to influence the results e.g. age, parity, education, BMI level etc. (Table 1).

Since we aimed for an in-depth understanding of the participants’ experiences, we developed an open semi-structured interview guide (additional file 1). This guide was continually updated as the participants provided perspectives that we wanted to explore further. The interviews provided data for two distinct manuscripts. The first study explored participants’ experiences and understandings of their weight history from childhood to motherhood in relation to their childhood quality [40]. Based on this information, in the present study, we examined participants’ experiences of maternity care provision in the context of their childhood experiences regarding higher weight, body, and general childhood quality. This context helped to illuminate the reactions and strategies that participants described in their encounters with maternity care.

The participants, all unfamiliar to the researchers, were interviewed by HLS, with interview experience from previous studies in maternity care. Interviews lasted 70–120 min and were conducted in the participants’ homes or a suitable room at the local university or hospital, based on their preferences. To ensure that the participants’ descriptions were understood as intended, ambiguities were clarified and the interviewer’s understanding and interpretations of the information were regularly summarized during the interview. HLS wrote reflection notes on each interview to document her overall impression and aspects of the participants experiences that were particularly surprising or notable. The interviews were audio recorded, anonymized and assigned with a pseudonym in connection with a slightly modified verbatim transcription, done manually by HLS. Participants were included until we judged that we had information power to answer the research questions in breadth and depth [42].

### Data analysis

We used reflexive thematic analysis to identify patterns of meaning across the dataset and develop themes through the six-phase process described by Braun and Clarke [43]. This methodological approach was chosen because it is not strictly categorized as either inductive or deductive, but is positioned between the two. Similarly, following Braun and Clarke, the themes developed from the analysis encompassed both descriptive and interpretative elements, often presenting a blend of the two [43, 44]. This flexibility was important as this dataset contributed data to two studies, where the results from the first study provided relevant information to understand the data in the present study [40]. The last steps of the analysis are underpinned by Løgstrup’s phenomenological theory, The Ethical Demand, as it highlights an important pattern in our data [45].

The interviews were initially read in depth by HLS and JH. HLS wrote extensive notes, using Braun and Clarke’s reflexive questions in the analysis [43]. These notes were developed into a document that was shared with JH, TTEN, LOG and EBM, together with an overview of codes and preliminary themes. In several meetings, we discussed the participants’ descriptions and experiences and developed the final themes. The final themes were formulated and written out by HLS and again shared with JH, TTEN, LOG and EBM for discussion. The coding process revealed that the interviews contained patterns and descriptions that made it relevant to read the interviews in light of Løgstrup’s phenomenological theory as presented in “The ethical demand” [45], where he explains factors that regulate human interaction. Løgstrup used the term “zone of the untouchable” to describe the boundary zone that regulates interpersonal interactions. This zone refers to the private space that people usually do not wish to share with others. Stepping into this zone is described as potentially disrespecting a person’s feelings and violating their integrity [45, 46]. Throughout the iterative process, a codebook was employed to document essential decisions and modifications. To safeguard the quality of the analysis, Braun and Clarke’s 15-point checklist was utilized, together with the consolidated criteria for reporting qualitative studies [43, 47].

## Results

Most participants in this study were in their thirties, married or cohabiting and had been born and raised in Norway. The majority reported a college and university educational level and worked full-time. Most of them had a pre-pregnancy BMI of ≥ 35 kg/m^2^. For baseline characteristics, see Table 1.

The results of this study comprised of an overarching theme supported by three main themes (Table 2).

**Table 1:** Socio-demographic characteristics of the participants (*n* = 14)

**Age**	
26–30	4
31–35	6
36–42	4
**Marital status**	
Married	5
Cohabiting	7
Single	2
**Country of birth**	
Norway	12
Other	2
**Place of residence**	
Urban	6
Semi-urban	3
Rural	5
**Educational level**	
Lower than high school	1
Certificate of apprenticeship	3
College or university	10
**Occupational status**	
Employed, full time	12
Employed, part time	1
Unemployed	1
**Parity**	
Primiparous	7
Multiparous	7
**Pre-pregnancy body mass index**	
Obesity class l (30- <35 kg/m^2^)	4
Obesity class ll (35- <40 kg/m^2^)	5
Obesity class lll (≥ 40 kg/m^2^)	5

**Table 2:** Overview of overarching theme with corresponding themes

Overarching theme Being pregnant with a high BMI: a vulnerable condition		
**Theme 1**	**Theme 2**	**Theme 3**
Loaded conversations: a balancing act	Dehumanization: an unintended drawback of standardized care	The ambivalence of discussing weight and lifestyle

### Being pregnant with a high BMI: a vulnerable condition

The participants had varied weight histories prior to their pregnancy, which affected their experiences with maternity care differently. Some individuals described having had no thoughts about their body and weight in the first part of their pregnancy. Hence, many described being caught off guard when confronted with having a body “outside the norm”, as their weight categorized their pregnancy as “high-risk”. Not being considered “good enough” due to their high BMI during pregnancy was described, while others expressed anger at not being seen as a whole person. Weight conversations with healthcare providers were described as intrusive and offensive when not approached in an open and empathetic manner.

#### Frida:

“*I wasn’t told I weighed too much until my first meeting with the midwife who was looking at her chart. Then I got really big mental problems. I’ve never felt so bad in pregnancy as I did after seeing that midwife….(.). I wasn’t good enough. I felt like she was looking down on me. I was fat.*”

Participants who experienced negative body awareness from childhood bullying and body criticism, as well as those who felt different due to their higher weight despite having a good childhood, were more prepared for weight-related discussions in maternity care. These women therefore employed strategies to assist them in handling weight-related discussions during interactions with healthcare providers. Those who had internalized that their weight was their own personal responsibility dreaded meeting new health care providers because they were afraid of being judged as irresponsible and selfish. Some felt that they did not deserve a pregnancy as much as women of normal weight.

#### Hilde:

“*I was so ashamed. I stayed at home a lot and didn’t want anyone to see me. I was ashamed talking to the staff because I felt selfish. Like a stupid selfish person who was overweight*,* and yes*,* I have been so extremely ashamed. Because I feel like it’s almost proof that you…at least for other people*,* that you haven’t thought about it (the weight) properly.”*

### Loaded conversations: a balancing act

This theme illustrates how the participants described the way they and several healthcare providers navigated conversations about weight to protect the woman’s integrity. Løgstrup characterizes such conversations as a “zone of the untouchable”, a boundary zone in which interpersonal interactions risk disrespecting a person’s feelings and violating their integrity [46]. To step into this zone is defined by him as potentially disrespectful towards a person’s feelings and integrity [46, 48]. The relationship between the pregnant woman with obesity and the professional was described by some participants as characterized by healthcare professionals having the power, due to their professional positions. Giving advice on weight based on maternity care guidelines was described as increasing this asymmetry. The subject of risk associated with obesity and significant weight gain during pregnancy was an emotionally loaded topic for several participants. Evidence-based maternity care (i.e., including information about the risks of obesity) was experienced as an emotional burden by these participants. The participants’ descriptions showed that their perceived zones of the untouchable varied according to context, between individuals and over time.

#### Bente:

“*Three years ago*,* I’d definitely have been offended if they’d offered follow-up and guidance regarding my weight after childbirth. But now I’ve got a different view about that. Now it’s quite ok. I don’t mind. Well*,* ok*,* I do have a few extra pounds*,* but I’m still active. But if it had been like I couldn’t participate in things*,* that would have hurt more*”.

Participants’ strategies to protect their integrity during clinical conversations about weight are described below, in addition to their experiences of the way healthcare providers also used communication strategies to prevent violations of their integrity.

The strategy “to get ahead of them” was used by some women to protect themselves from weight bias, stigma and shame. For these participants, initiating conversations about weight before providers raised the concern served as a way to get ahead of their weight-related shame. Providers’ evasive talk about weight instead of clear and considerate conversations was described in negative terms by some participants. Vague talk about weight turned the topic into a taboo.

#### Hilde:

“I want to get ahead of them *because I don’t want it (conversations on weight) to be awful for them to ask about my weight or find out if I know it’s not good for me. I don’t want to put other people in a position where they think it’s horrible to have to start asking about the weight of someone who’s obviously very overweight.*”

At the prenatal check-ups, some participants talked about their weight problems and the reasons for these, their attempts to lose weight and how the problems affected them during pregnancy and in life generally. The women wanted to demonstrate both their awareness that weight was considered a health challenge and that they were taking steps to promote their own health. In this way some women were protecting themselves from unpleasant conversations that could lead to feelings of shame and guilt. Although most of them were used to speaking up and expressing their opinion in other contexts, they could fall silent in conversations related to their weight due to shame. By informing healthcare providers about their weight experiences, several found that the professionals gained a greater understanding of their situation.

#### Grete:

*“I told the midwife I’d been for check-ups because of my weight. I talked quite a lot about what I’d been doing this past year and I think that was a good idea because then I like put my cards on the tabl*e. So that’s the way it is, I said, and now I’m going out for a walk every day. *And she thought that was very good. She said I’d done well.*”

However, some participants found that the strategy “to get ahead of them” did not help.

#### Kristine:

“*I tried to explain that it’s not because I just laze about all the time. I’m actually sick. I have an illness. But they won’t believe you when you’re overweight. I said several times that I don’t actually sit around digging into a box of chocolates all day*,* but I still look like I do.*”

The participants*’* descriptions of their encounters with healthcare providers in maternity care show that midwives and doctors also used strategies to protect the woman’s integrity. They described how some professionals realized that weight was a private matter and a charged topic for many. Approaching the subject of weight in that matter was perceived as respectful, helping the participants to tell their weight story. Some felt they could participate in shared decision-making regarding how much weight and lifestyle to include in the check-ups. For many of the women it was important that weight was not the main issue.

#### Elise:

“*My doctor noted down my weight and said: Your BMI is 33*,* so now I ought to say that you shouldn’t gain much weight during your pregnancy*,* but you’re grown up*,* so I don’t feel I should interfere too much. Then I asked if there was anything I should watch out for regarding my weight.*”

The interviews revealed that a number of healthcare providers emphasized aspects of the women’s health that reduced the risk of complications, such as normal blood pressure and cholesterol and blood sugar levels.

Some participants expressed reluctance to be weighed during maternity check-ups however; they noted that the weighing became more acceptable to them when healthcare providers normalized their situation. Participants also found it helpful to be reminded that pregnancy was not the right period for dieting, and to hear that all pregnant women, regardless of weight, benefit from a healthy lifestyle. Some were reassured when healthcare providers emphasized that weighing provides essential information about both weight gain and loss, making it valuable regardless of baseline weight. Offering women the opportunity to weigh themselves at home was seen as a means of reducing shame and increasing their autonomy.

#### Anna:

“*She (provider) wanted to see my weight*,* but we didn’t need to talk about it. She said*,* ‘But I want to see that you’re putting on weight. Because it’s not a good sign if you’re not.’ So I had to be honest and say that I don’t want to focus on my weight because I think that’s hard. It hurts my feelings. She fully understood and respected that.*”

In many cases, healthcare providers paid scant attention to the woman’s weight in maternity care. This often happened when the women went for a check-up to a doctor who knew their history. These women assumed that prior knowledge of their weight history contributed to less need to address their weight. Some also suggested that the reason for not addressing their weight could be due to fear of harming the patient-doctor relationship. One participant reflected on why her weight was not mentioned at the check-ups:

#### Inga:

“*I think it was kind of because I had such a good relationship with my doctor. She knew a lot about me. I went to her when I was doing a program for weight reduction. So she knew me very well.*”

### Dehumanization: an unintended drawback of standardized care

Standardized care follows guidelines for addressing weight and lifestyle when pregnant women have obesity. The participants met many different healthcare providers at their pregnancy checks, which meant many first encounters where their high weight was the topic. Several participants felt that healthcare providers lacked knowledge and communication skills to handle the topic. It was described as a routine conversation without value for the participants because no time was set aside for an open and exploratory approach to discover the reasons for their weight problems. Several found that the professionals used the table for recording BMI to provide information about how much weight they could gain in pregnancy.

#### Frida:

“*She starts going through a form* [health card for pregnancy], *then she says: You’re not allowed to gain more than six kilos based on your weight. And you mustn’t eat that and that. You have to eat this and this. She doesn’t even look at me. And all the information she mentioned*,* it was what’s on* the“ *Health Norway”[the official website for information about and access to health services for residents of Norway] which I’d read many times before I went to her. Then we were done. When I left there*,* I thought: What happened? She really knew how to put me down*,* because she told me what I was and wasn’t allowed to eat*,* so I wouldn’t put on weight…(.). They have a list to tick off things*,* which they just read out*,* but there’s no chance of adapting things to different people.*”

Despite not having their diet assessed, most participants were instructed to avoid soda, candy, and snacks. Several participants perceived this as an assumption that pregnant women with obesity lacked knowledge and led unhealthy lifestyles; it was seen as provocative and unfair. Further, some found that healthcare providers asked about weight and lifestyle to the neglect of other topics that the participants felt were more important. Challenges with mental health was a topic that some felt should have been addressed instead of weight talk, but which several described as the cause of their weight problems.

#### Grete:

*“It was six weeks later. She’d received papers saying that I’d had a pretty hard time after giving birth* [cries]. *I’d just got down from the gynecological examination chair. Then the best she can do is to ask if I’ve started to change my diet. Then I thought*,* hey*,* you got the papers about how I felt after the birth.*”

Some healthcare providers were perceived as being provoked by the participants*’* weight. Some women described dehumanizing encounters with certain professionals. They described these experiences in great detail, which suggests that these encounters led to feelings of shame, guilt and inferiority. Several participants confirmed this. Some stated that certain professionals felt obliged to inform the women about the consequences of severe obesity in pregnancy without considering the impact of their information.

#### Kristine:

“*She said now let’s check your weight. Then she went and got some scales that didn’t go up far enough. So there was just an error in the display*,* and then she said*,* well*,* with your body you won’t be able to give birth in the natural way. You have put yourself*,* the baby and the maternity care in a very difficult situation by getting pregnant. Your baby will be huge. You have a high risk of premature birth and then it’s all because of your body.*”

With some participants, the healthcare providers used loaded words to emphasize the seriousness of their weight, which in some lead to mistrust. The women wondered whether the risk was really as high as it was presented.

#### Bente:

“*I still don’t know why there was so much monitoring actually. I still don’t know what that risk was. Was it as big (risk) as they suggested or was it just hype?*”

The consequence of healthcare providers violating the women’s zone of the untouchable was that they lost the opportunity to enter into a dialogue about weight and lifestyle, because the participants then changed their healthcare provider or complained about their maternity care. Those who continued to have check-ups with someone they had complained about stated that weight and lifestyle were then no longer discussed. Women who went to a new doctor or midwife told them about their previous negative experience of conversations about weight. This prevented new conversations about weight and lifestyle. Subsequent pregnancies were also affected because they did not want to discuss their weight due to their negative experiences from the previous pregnancy. The consequences of a standardized, insensitive conversation about weight and risk therefore appear to prevent professionals from providing good health promotion and prevention advice in maternity care because they are often only given one chance to talk about weight.

#### Elise:

“*I didn’t feel like asking questions. I didn’t feel like going back to the midwife afterwards. So that was the last time I saw a midwife. I saw the doctor for the rest of my check-ups.*”

### The ambivalence of discussing weight and lifestyle

The participants all had additional check-ups by specialists because of their weight, as recommended by Norwegian guidelines. At the check-ups, several were offered professional weight counseling, either during pregnancy or after the birth. This offer was well received by most of them, and it was generally the first time anyone had offered them professional help with their weight problems.

#### Cecilie:

*“Someone asked about referring me to professional weight monitoring. I said I’d like that very much. I felt really relieved that now I’d talk to someone who could maybe help me or guide me on the right path. I’m very glad they did this. But when you’re pregnant*,* you shouldn’t lose weight. So there’s not much you can do just then.*”

Although several participants had a good experience of conversations about weight, ambivalence about the attention given to weight in their maternity care was described. Under this theme, we first describe what the participants felt was important for weight and lifestyle conversations to be useful to them. Then we describe the ambivalence many participants felt about these conversations and why several wanted most of the guidance to take place after the birth, although the initiative should be taken during pregnancy.

The interviews reveal that healthcare providers should have an open, tolerant and person-centered attitude for conversations about weight to be experienced as meaningful. They emphasized that it was the attitude of some professionals that upset them, not the fact that their weight and lifestyle was addressed. Healthcare providers were encouraged to demonstrate commitment by dedicating time for weight conversations and having a thorough understanding of the topic. Participants recommended initiating a dialogue with unbiased, open-ended questions to encourage women to share their individual perspectives on weight and lifestyle.

#### Johanne:

“*I felt like it was something she talked to everyone about. So I didn’t feel there was anything abnormal about me. We had a good talk.*”

Several participants emphasized that not everything needed to be said directly or related to weight, and preferred the message to be “wrapped up a bit” because it hurts to be bombarded with weight-related risks. If the professional wants to be direct, this must be done in a neutral and considerate manner.

#### Hilde:

“*Just saying things in a slightly different way can mean a lot. I have high blood pressure too. I know that blood pressure is related to weight. But no one’s said to me: ‘You must realize that you have high blood pressure because you’re so overweight.’ My doctor says something like: ‘Well*,* then let’s hope you can stop taking the blood pressure medicine after a while*,* because blood pressure often goes down when your weight goes down.’ He says it in such a nice*,* matter-of-fact way.*”

The reasons for the women’s ambivalence were complex. Some of them pointed out that pregnancy gave them some balance, i.e., they were in a good period of their lives. An emotional balance helped them to maintain a healthy lifestyle. Others stated that they had spent years finding a lifestyle that suited them. They were afraid that a strong focus on weight and lifestyle would disturb the balance they had found, especially if they felt judged and misunderstood by healthcare providers. Despite some women not desiring a lifestyle focus themselves, they believed that maternity care providers should offer lifestyle advice to pregnant women who clearly are in need of it.

#### Anna:

*“Some people may need a bit more advice and support to get on the right track. You can often tell from the person. If she’s doing all the wrong things and needs some advice about what would be sensible…(…). Because I was so big*,* the doctor wondered if I wanted to see a nutritionist. I said no*,* because I don’t want to*,* I know what works for me*,* what I enjoy doing*,* so I don’t need that.*”

If weight is to be addressed during pregnancy, the woman must find it important and useful, according to the participants. Several of them thought it was important to offer professional weight counseling and monitoring postpartum, which they considered a good reason and time to address weight. Weight and lifestyle conversations that the participants found unhelpful or unnecessary were one of several factors that made them ambivalent about this aspect of maternity care.

The participants*’* ambivalence was also linked to previous experiences of dieting and weight reduction. Several had lost some weight previously but were unable to maintain their weight loss. Lifestyle changes worked when they had time for exercise and a healthy diet, but it was difficult in a busy life, and more so with motherhood. However, some felt that their focus on motherhood motivated them to make gradual lifestyle changes because they achieved routines and balance in their lives. Others were content with their current situation and saw no reason to discuss weight. Still, for most participants, pregnancy was regarded as a special period in life that they wanted to enjoy, which conflicted with significant lifestyle changes that reminded many of dieting.

As dieting is not considered safe during pregnancy, many participants expressed that it is better to offer enhanced weight counseling postpartum. Other factors that make it difficult to follow lifestyle guidance are periods of vomiting and nausea and generally reduced physical fitness in pregnancy.

#### Dina:

“*She* [the nutritionist] *had very little understanding of pregnancy*,* poor appetite and nausea. I don’ t think she has children of her own*,* or that she’ has had morning sickness like this herself. So talking to her wasn’t much use.*”

## Discussion

This study aimed to explore the experiences of pregnant women with obesity in encounters with healthcare providers in maternity care. Overall, our findings reflect the challenge of entering maternity care with obesity, especially for women unprepared to be seen as outside the norm.

The first theme, “Loaded conversations: a balancing act”, emphasizes how pregnant women with a history of body criticism or obesity-related otherness proactively protect their “zone of the untouchable”, using strategies to gain some control and reduce the risk of weight stigma. Participants also described helpful healthcare providers’ strategies in managing the professional relationship, protecting them from feelings of intrusion and violation. The second theme, “Dehumanization: an unintended drawback of standardized care”, highlights the pitfalls of prioritizing standardization over person-centered care. Finally, the third theme, “The ambivalence of discussing weight and lifestyle ”, describes women’s underlying ambivalence towards current weight practices in maternity care.

Women with pre-pregnancy obesity expressed feelings of shame, inferiority and frustration due to weight stigmatizing encounters with health care providers. Not feeling “good enough” as a pregnant mother in the encounters with weight- and risk-focused maternity care was described by several of the participants. Nyman et al. have previously described similar findings and related them to the tendency of healthcare providers to emphasize the physical aspects of pregnancy care, leaving little space for womens’ own perspective on weight [26].

In “The ethical demand”, Løgstrup explains the conflictual interaction between people where public morality, laws, rules and guidelines do not always tell us how to act in various situations [45]. The zone of the untouchable is the private space that we do not want to share with others; our awareness of this inviolable space determines interpersonal interaction [46, 48, 49]. Theme 1 in this study showed how both patients and healthcare providers navigated the untouchable zone as they addressed weight, lifestyle and risk. Openness of speech is necessary to prevent isolation in the zone of the untouchable; when speech is not open, we may find interpersonal interaction intolerable because of what is unspoken [46, 48, 50]. In this study, several participants said that their weight was not mentioned in maternity care, which they assumed was because the professionals were afraid of offending them and harming the professional relationship. This finding has been demonstrated in previous studies [18]. Keenan et al. reported that pregnant women with obesity often found little or no focus on their weight in maternity care [51]. Findings from a recent scoping review by Dieterich and Demirci corroborate the insufficient focus on weight management in women with pre-pregnancy obesity. Some studies included in this review suggested that pregnant women underestimate the importance of pre-pregnancy obesity and weight gain during pregnancy for both the unborn child and their own health, due to a lack of communication from their healthcare providers [18]. Late, vague and inconsistent weight communication from healthcare providers are contributing to missed opportunities for health promotions [18]. Studies have also found that many healthcare providers do not know how to talk about weight, which reflects a situation of powerlessness where the professional knowledge and experience of healthcare providers is inadequate [15, 18, 51–53]. Motivational interviewing (MI) was described as a useful approach when addressing difficult topics in maternity care. Training in motivational interviewing helped healthcare providers expand their awareness of communication, to connect better with pregnant women, and become more mindful of the importance of listening and asking for permission rather than giving advice [54].

Weight stigma increases the risk of poor health [55]. The beliefs about weight that patients and clinicians carry into clinical encounter harm patient-clinician communication and clinical effectiveness both in the commission and omission of language. When healthcare providers don’t know the boundaries of the zone of the untouchable they run the risk of not understanding the effect of their words or their silences. As described by the participants, this effect appears to be an obstacle to consistent health prevention work with this group of pregnant women, who often change healthcare providers due to weight stigma. Change of healthcare providers can hinder women from building trustful relationships with their healthcare providers. Such trust is crucial for women to seek help for worries and symptoms during pregnancy and beyond. Discontinuity in care can delay the detection of complications, risking harm to both mother and child [56]. Inconsistent maternity care thus represents a double burden forthese women: not only a lost opportunity for health promotion, but also the effect of weight stigma on their future health.

We found that some participants facilitated open discussions about their weight, employing the strategy “to get ahead of them,” to protect themselves against stigma and shame, and to facilitate open talk about their weight. Shame anxiety has been described as a fear of being objectified, judged, labeled, and rejected by others, which can make people use strategies to avoid potential shaming situations in line with our findings [57]. Getting a head of them, may be understood as “shame avoidance”, and is found to be commonly employed by individuals who experience chronic shame [57]. Some women described some of the encounters in maternity care up to be respectful of their stories indicating room for a “shame-sensitive practice” in healthcare [57]. Shame avoidance in this study was particularly described by women with early and significant body criticism or expressed perceived otherness due to their body weight. By understanding and acknowledging the use of the “to get ahead of them” strategy as a defensive reaction of shame, healthcare providers can gain insight into the importance of shame. Hence, sensitivity to pregnant women’s ACEs and previous encounters with weight-related bias is essential to good care.

Similarly to findings from a review on the impact of perceived weight bias in encounters with healthcare providers, our findings suggest that weight stigma and dehumanization prevented professionals from providing continuous care because of the need for women to change their provider [58]. A study involving 20,000 insured US adults found that individuals with obesity had 52% higher odds of changing healthcare providers than those with normal weight [59]. Moreover, in another US study, Rodriguez et al. discovered that almost 8% of pregnant women switched healthcare providers due to weight stigma, aligning with findings from our study [29]. A large British study investigating the association between maternal BMI and access to maternity care found a slightly increased likelihood of delayed access for women with pre-pregnancy overweight or obesity [60]. These findings are supported by similar studies in the general population [58, 61]. Delayed access to both antenatal and postpartum care negatively impacts this group of pregnant women, since both late initiation of antenatal care and a high pre-pregnancy BMI increase the risk of adverse outcomes for both the mother and her child [60]. In contrast, our participants did not describe lower or delayed use of healthcare services. This may be because pregnant women feel responsible for the life of the unborn child in addition to their own health, while maternity care in Norway gives dissatisfied women the possibility to change their healthcare provider rather than simply not attending check-ups. This assumption is supported by a qualitative study from the US, where several pregnant women reluctantly attended antenatal check-ups, but skipped postpartum care if they felt badly treated by healthcare providers [62].

In line with the findings in this study, previous studies examining the preferences of pregnant women with obesity in relation to weight monitoring, risk assessment and lifestyle guidance emphasize the need for healthcare providers to acknowledge the patient’s view of contributing factors to obesity, show interest in patients’ backgrounds, embrace a non-judgmental approach, and refrain from giving unsolicited advice [11, 26, 63]. In contrast to several similar studies, we found that many women were ambivalent about professionals providing enhanced lifestyle guidance during pregnancy [52, 64, 65]. The participants in our study were interviewed about their weight history and childhood quality in addition to their experiences of maternity care. This may have increased their awareness of the similarities between their maternity care experiences and the influence of their childhood on their weight development. Confidential conversations about childhood and weight may also have increased the participants’ confidence to express their honest opinion about the approach to weight in their maternity care.

As in previous research, our third theme, “The *ambivalence of discussing weight and lifestyle*”, suggest that pregnancy can be an important period to identify women who want further weight advice and monitoring, but that this should be offered postpartum [63]. Olander and Atkinson have described the difficulty in recruiting pregnant women with obesity to programs to reduce weight gain during pregnancy [66]. Similarly to our results, the women mentioned barriers such as nausea, reduced physical fitness, and a desire not to focus strongly on weight during pregnancy. A lack of motivation and interest has also been found to affect participation in such programs, in addition to more practical barriers [66]. A Danish study of pregnant women’s experiences with increased weight guidance in a retrospective perspective found that the participants had not maintained the lifestyle changes over time [67]. In contrast to the results in this and other studies suggesting that the postpartum period is preferable for enhanced lifestyle guidance [68], pregnant women with obesity often find that their problems, contributing to weight challenges like for example emotional problems, eating disorders or complications related to obesity, are ignored after the birth, despite an in-depth focus on weight and risks during pregnancy [63, 69, 70]. The postpartum period is suggested to be a time of increased body dissatisfaction, associated with disturbed eating patterns, increased weight, and worse mental health [71]. Offering postpartum lifestyle and weight follow-up can contribute to these adversities, along with additional pressure on women to return to their pre-pregnancy bodies or achieve a normal weight, which may neither be desirable nor possible. Lifestyle counseling should therefore focus on the health benefits of following dietary and activity guidelines for the general population, rather than weight itself.

### Strengths and limitations

A major strength of our study is the inclusion of contextual information about participants’ interpretations of their weight history from childhood to motherhood, along with information on childhood quality. This information proved crucial to understanding their descriptions and interpretations of healthcare encounters during pregnancy in relation to weight and risk management. The diverse professional backgrounds of the research team enabled us to examine the data from different perspectives. Additionally, the project design involved strong service user engagement from three key interest organizations. We obtained a strategic sample with a wide range of important baseline characteristics, including age, marital status, parity, education level, and various degrees of obesity. Although most participants were well educated, their experiences did not appear to differ significantly from those with a lower educational level. However, we only recruited one participant born outside Europe, which may have limited the information power regarding ethnic diversity.

## Conclusion

This study shows that standardized weight monitoring and advice in maternity care put these women in a vulnerable position, in contrast tothe emotionally supportive care women with obesity say they need. Learning from these women’s experiences and their need for neutral, non-judgmental communication to protect their integrity highlights the importance of patient-centered practices and shame-sensitive care. An open mindset toward the insider perspective of pre-pregnancy obesity, as opposed to standardized care, can create a safe space for health promotion. For pregnant women and their offspring, weight stigma and the avoidance of discussing weight by professionals can result in missed opportunities to positively impact their future health. Improving communication skills and staying updated with knowledge can be a significant advantage for healthcare personnel to effectively care for this group of pregnant women in a respectful and considerate manner. Future research may examine how maternity care providers can establish enhanced postpartum weight and lifestyle guidance that meets the wishes expressed by several participants in this study.

### Electronic supplementary material

Below is the link to the electronic supplementary material.

Supplementary Material 1

Supplementary Material 2

Supplementary Material 3

## Data Availability

The datasets analyzed during the current study are not publicly available because the participants have not consented to sharing the interview material.

## Abbreviations

ACEs: Adverse Childhood Experiences
BMI: Body Mass Index
MI: Motivational interviewing
