# Supplementary material for: Navigating weight, risk and lifestyle conversations in maternity care: a qualitative study among pregnant women with obesity
Source: BMC Pregnancy Childbirth. 2024 Aug 23;24:552. doi: 10.1186/s12884-024-06751-1 (PMC11344406; doi:10.1186/s12884-024-06751-1)
Supplement: Supplementary file 1 — Supplementary Material 1 [file 12884_2024_6751_MOESM1_ESM.docx]

**Introduction to the interview:**

- *Information about confidentiality in interviews. All data will be anonymized and stored securely in accordance with approved guidelines.*
- *You can decide yourself how much information you want to share.*
- *You can interrupt the interview at any time for a break or to terminate it. (Agree on signs for the woman to give when she needs a break or wants to end the interview)*
- *I may take notes during the interview or take short breaks to think of natural follow-up questions to ask you.*
- *The interview may take around 60-90 minutes.*
- *There are no right or wrong answers, but it is important that you talk about your personal experiences as you have experienced them yourself. Please give examples.*

**Topics for part two of the interview: weight bias, what are the women's experiences of prenatal care when they have a high BMI?**

1. Please tell me about your pregnancy care and meeting healthcare staff during your pregnancy.

2. What was your experience of how your weight affected your pregnancy care?

3. Pregnancy care has been described as a window of opportunity where it is easier for pregnant women to make positive changes to their lifestyle. What do you think about this idea?

4. How was this for you?

**Topics that stood out during several interviews. These topics should be introduced into the conversation if the women do not bring them up themselves.**

- Attitude towards and expectations for pregnancy care
- Action strategies and defense mechanisms

5. Are there any other experiences that you think are important to talk about in this interview that I have not mentioned? Please tell me about them.
