# Supplementary material for: Navigating weight, risk and lifestyle conversations in maternity care: a qualitative study among pregnant women with obesity
Source: BMC Pregnancy Childbirth. 2024 Aug 23;24:552. doi: 10.1186/s12884-024-06751-1 (PMC11344406; doi:10.1186/s12884-024-06751-1)
Supplement: Supplementary file 2 — Supplementary Material 2 [file 12884_2024_6751_MOESM2_ESM.docx]

**Date:**

**First name:**

**Age:**

**Place of residence:**  Rural  Semi-urban  Urban

**Marital status:** Married  Cohabiting  Single

**Highest completed level of education:** Primary/lower secondary  Upper secondary Vocational qualification  Higher education

**Work/job:**

**Full- or part-time (when not on maternity leave):**

Full-time Part-timeOther

**Weight at start of pregnancy:**

**Who gave you pregnancy check-ups?** MidwifeYour doctor  Combination

**Height:**

**Number of children:**

**Delivery method:** Normal delivery Vacuum/forceps  Planned cesarean  Unplanned cesarean

Emergency cesarean

**How many weeks pregnant were you at delivery?**

**Feeding:** Breast-feeding: Yes  No  Number of months of breast-feeding Bottle-feeding: Yes  No

Combination of breast- and bottle-feeding

**Please add any comments here:**
