# Supplementary material for: Navigating weight, risk and lifestyle conversations in maternity care: a qualitative study among pregnant women with obesity
Source: BMC Pregnancy Childbirth. 2024 Aug 23;24:552. doi: 10.1186/s12884-024-06751-1 (PMC11344406; doi:10.1186/s12884-024-06751-1)
Supplement: Supplementary file 3 — Supplementary Material 3 [file 12884_2024_6751_MOESM3_ESM.docx]

| **Table A3: Codes, sub-themes and themes** | | |  |
| --- | --- | --- | --- |
| **Codes** | **Sub-themes** | **Themes** | **Overarching theme** |
| - Sweeping the issue under the rug - weight, risk and lifestyle left unaddressed - Protecting the professional relationship - Dancing around the issue - Being one step ahead of healthcare providers/putting your cards on the table (the women’s protective strategy) - Strategies to protect the woman’s integrity (not entering the untouchable zone) - Respect, interest and openness - Between ethics and guidelines - Creating a balance, including emphasizing what is normal | - Balancing along the zone of the untouchable | Loaded conversations: a balancing act | **Being pregnant with a high BMI: a vulnerable condition** |
| - Defined by your weight and given a label - Being a burden - Encounters that encroach upon the untouchable zone: the means justify the ends - No assessment of the woman’s life situation, straight to the intervention (weight bias) - Loaded words about a touchy subject and a risk focus to bring about change - The message may be acceptable, but the wrapping is all wrong - Failing to realize the effect of one’s words (poor communication skills) - The health card as the standard opener of conversations about weight - Without value/shouldn’t be pregnant/different - Bad timing - talk about weight has priority over more important topics - You only have one chance, or the woman will keep quiet or find another healthcare provider - Limited space for health promotion | - In the shadow of the scales: standardized, quantified care - Standardized care meets life stories | Dehumanization: an unintended drawback of standardized care |  |
| - Respectful, interested, open and adaptable - Good knowledge of lifestyle guidance and good communication skills - Dedicating time for conversations about weight - Going to have a baby - lead a healthy life - Prenatal care: a critical gaze can trigger an eating disorder - Offers of specific lifestyle guidance that the women find useful - Barriers in pregnancy - Postpartum care is most important | - We may discuss weight, food, and lifestyle - but on my terms - I’m open to discussing my weight and lifestyle, but… - the ambivalence | The ambivalence of discussing weight and lifestyle |  |
|  |  |  |  |
